# Efficacy and safety of triplet chemotherapy plus anti-EGFR agents in metastatic colorectal cancer: a systematic review and meta-analysis

**DOI:** 10.1186/s12957-022-02707-x

**Published:** 2022-08-15

**Authors:** Qian Wu, Huan Wang, Suqin Zhang, Yifei Zeng, Wei Yang, Wenjun Pan, Guodai Hong, Wenbin Gao

**Affiliations:** Department of Oncology, Shenzhen Luohu People’s Hospital, Shenzhen, Guangdong 518000 People’s Republic of China

**Keywords:** Metastatic colorectal cancer, Anti-EGFR, Triplet chemotherapy, Cetuximab, Panitumumab

## Abstract

**Background:**

To date, the optimal treatment for potentially resectable metastatic colorectal cancer (mCRC) patients has yet to be determined. Encouraging results have been reported in studies exploring the efficacy of triplet chemotherapy plus anti-epidermal growth factor receptor (anti-EGFR) target agents. Thus, we conducted a meta-analysis to evaluate the efficacy and safety of triplet chemotherapy plus anti-EGFR target agents.

**Methods:**

We systematically searched the PubMed, Embase, and Web of Science databases from December 2004 to October 2021 for studies examining the efficacy of triplet chemotherapy plus anti-EGFR target agents in mCRC patients. The primary outcomes were the objective response rate (ORR) and R0 resection rate (R0RR), and the secondary outcomes were median progression-free survival (mPFS), median overall survival (mOS), and toxicity. Data were analyzed with R software 4.1.2.

**Results:**

Fourteen studies comprising 762 patients with mCRC were included in this meta-analysis. Analysis with a random effects model revealed that after treatment with triplet chemotherapy plus anti-EGFR target agents, the pooled ORR was 82% (95% CI= 76–88%, *I*^2^= 76%), and the pooled R0RR of colorectal liver metastasis (CLM) was 59% (95% CI= 49–68%, *I*^2^= 60%). The mPFS ranged from 9.5 to 17.8 months, and the mOS ranged from 24.7 to 62.5 months. A total of 648 grade 3 or 4 adverse events were reported; the most commonly reported events were diarrhea (174/648), neutropenia (157/648), and skin toxicity (95/648), which had pooled prevalence rates of 29% (95% CI= 20–39%, *I*^2^= 84%), 28% (95% CI= 20–37%, *I*^2^= 77%), and 17% (95% CI= 11–24%, *I*^2^= 66%), respectively.

**Conclusions:**

Triplet chemotherapy plus anti-EGFR agents therapy seems to be capable of increasing the ORR of mCRC patients and the R0RR of CLM patients. The toxicity of this treatment is manageable. High-quality randomized controlled trial (RCT) studies are required for further validation.

**Supplementary Information:**

The online version contains supplementary material available at 10.1186/s12957-022-02707-x.

## Background

Colorectal cancer is the third most common malignancy and the second leading cause of cancer-related deaths worldwide. Approximately one-quarter of patients are unresectable or have metastatic colorectal cancer (mCRC) at the initial diagnosis [1]. The 5-year survival rate associated with mCRC is less than 20%, whereas the number is more than 80% in early-stage CRC patients [2].

With recent progress in cancer management, the median overall survival (mOS) of mCRC has widely improved from approximately 6 months (best supportive care) to nearly 30 months after standard systematic treatments in the past 20 years [3]. In addition to new drugs and novel technologies, these improvements are mainly attributed to biomarker-based patient selection and effective therapeutic combination strategies [4, 5]. The first-line therapy plays a critical role in the successful treatment of patients with mCRC, especially for patients with potentially resectable metastases. Triplet chemotherapy, the combination of 5-fluorouracil, irinotecan, and oxaliplatin [2, 6], has been proven to be effective and tolerable in pancreatic cancer with characteristics of rapid tumor shrinkage and improved secondary surgery rate since 2010 [7–9]. This regimen is also gaining importance in the treatment of mCRC. The randomized, controlled, phase 3 TRIBE study compared FOLFOXIRI plus bevacizumab to FOLFIRI plus bevacizumab and showed a better objective response rate (ORR; 65 vs. 54%, *P*<0.05) and mOS (29.8 vs. 25.8 months, hazard ratio [HR] 0.8, 95% confidence interval [CI] 0.65–0.98, *P*=0.03) in the triplet plus bevacizumab arm without increasing intolerant toxicity [10]. This finding led to major guideline recommendations for this therapy as a first-line treatment for mCRC patients. Anti-epidermal growth factor receptor (anti-EGFR) target agents (e.g., cetuximab or panitumumab) also proved to have a high response rate in mCRC treatment. The FIRE-3 trial established that in comparison with FOLFIRI plus bevacizumab, a combination of FOLFIRI and cetuximab improved the ORR (77 vs. 65%, *P*=0.014) and mOS (33 m vs. 26 m, HR=0.75, *P*=0.011) in rat sarcoma (RAS) gene wild-type patients with left-side disease [11]. Since multiple studies revealed the advanced efficacy of both FOLFOXIRI and anti-EGFR agents (in RAS and BRAF wild-type patients) [12–14], one question was raised: in RAS and BRAF wild-type mCRC patients, will the combination of triplet chemotherapy and anti-EGFR agents improve the ORR or secondary resection rate? Although this regimen is not recommended in major guidelines, there are an increasing number of clinical trials exploring its efficacy and safety [15]. The POTHER trial, the first phase II prospective trial aiming to assess the effectiveness of cetuximab plus triplet chemotherapy in colorectal liver metastasis (CLM), achieved an ORR and secondary R0 resection rate (R0RR) of 79.1% and 60%, respectively, with tolerable toxicity [14]. The VOLFI trial compared the efficacy of panitumumab plus mFOLFOXIRI with FOLFOXIRI alone in 96 patients with RAS wild-type unresectable mCRC randomized into two groups. The ORR in the panitumumab plus mFOLFOXIRI group was significantly higher than that in the FOLFOXIRI group (87.1 vs. 60.6%, odds ratio [OR]: 4.469, 95% CI 1.614–12.376, *P*=0.0041). However, despite the encouraging results, the majority of the studies were single-arm or retrospective studies, and thus, definitive conclusions about the efficacy and toxicity of anti-EGFR plus triplet chemotherapy could not be drawn.

It is generally acknowledged that meta-analysis is a powerful statistical tool to overcome the limitation of different sample sizes from individual studies and to generate the best estimation. Therefore, we conducted a meta-analysis of all eligible published studies to evaluate the efficacy and safety of triplet chemotherapy plus anti-EGFR agents in treating mCRC patients as a first-line regimen.

## Methods

This systematic review and meta-analysis is registered in the PROSPERO database (CRD42021289370, https://www.crd.york.ac.uk/PROSPERO/). It was performed following the meta-analysis (PRISMA) guideline (details were presented in Additional file 1).

### Search strategy

We systematically searched the PubMed, Embase, and Web of Science databases for relevant studies published from December 2004 (date of cetuximab approval by the Food and Drug Administration [FDA]) to October 2021. Additionally, abstracts from the American Society of Clinical Oncology (ASCO) annual meeting (December 2004 to 2021), the European Society of Medical Oncology annual meeting (2004–2021), and the World Congress on Gastrointestinal Cancer (2004 to 2021) were screened. Reference lists of included studies were screened to identify other eligible studies. Abstracts from the meetings were searched through the meetings’ official websites to identify relevant citations.

Search strategies included the following:

The PubMed search terms were as follows:

- ((Colorectal Neoplasm) OR (Colorectal Tumor) OR (Colorectal Cancer) OR (Colorectal Carcinoma)) AND (Cetuximab OR Erbitux OR (IMC C225) OR (MAb C225) OR (C225) OR panitumumab OR (Human Panitumumab Antibody) OR (ABX-EGF MAb) OR (ABX EGF Monoclonal Antibody) OR Vectibix OR anti-EGFR) AND (FOLFOXIRI OR FOLFIRINOX OR (triplet chemotherapy))

The Embase search terms were as follows:

- (‘colorectal neoplasm’ OR ‘colorectal tumor’ OR ‘colorectal cancer’ OR ‘colorectal carcinoma’) AND (‘c225’ OR ‘erbitux’ OR ‘imc 225’ OR ‘ly 2939777’ OR ‘Cetuximab’ OR ‘abx egf’ OR ‘panitunumab’ OR ‘vectibex’ OR ‘vectibix’ OR ‘anti-EGFR’) AND (‘FOLFOXIRI’ OR ‘FOLFIRINOX’ OR ‘triplet chemotherapy’)

Search terms for the Web of Science were as follows:

- (Colorectal cancer) AND (cetuximab OR Erbitux OR (C225) OR panitumumab OR Vectibix OR anti-EGFR) AND (FOLFOXIRI OR FOLFIRINOX OR (triplet chemotherapy))

### Inclusion criteria

1. Diagnosis: The studied patients were diagnosed with metastatic colorectal adenocarcinoma and pathologically confirmed.
2. No restriction existed for patient racial/publication status (full text or meeting abstract) as long as data were completed.
3. Patients were treated with triple chemotherapy (5-fluorouracil, irinotecan, and oxaliplatin/capecitabine) plus anti-EGFR (cetuximab or panitumumab) agent as a first-line chemotherapy.
4. At least three of the treatment outcomes mentioned below were reported.
5. The study design included RCTs, prospective nonrandomized trials, and observational studies (prospective or retrospective) published from December 2004 to October 2021 with a sample size of at least 10 patients.

### Exclusion criteria

1. The studies were not written in English.
2. All patients in the study had a RAS or BRAF mutation.
3. The studies contained incomplete data on the outcome of interest.
4. Studies with duplicate data or report analysis.

### Data extraction

All candidate articles were evaluated and extracted by two independent authors (H Wang and SQ Zhang). If disagreement occurred, a third author (Q Wu) was consulted. Data were extracted from the eligible studies using a standardized extraction form.

For each study, the following data were extracted: first author, year of publication, country, study period, median age, total number of cases, sex ratio, study design, treatment strategy, RAS status, BRAF status, ORR, number of liver-limited patients, R0 resection rate (R0RR), mPFS, mOS, follow-ups, and grade 3/4 adverse effects. The characteristics of the selected studies are shown in Table 1.

**Table 1:** Summary of the study characteristic

Author/year	Country/period of study	Trial name	Study design	Total patients	Gender(M/F)	Median age (years, range)	Primary location	RAS wt/mt/unknown	BRAF wt/mt/unknown	Primary endpoint	ORR (%)	mPFS (month)	mOS (month)	CLM/R0RR in CLM	mfollow-up (month, range)	MINORS
C Garufi [14]^*^/2010	Italy/2006–2008	POCHER	Pros-, case series	43	27/16	61 (33–75)	34/9^a^	30/7/6^d^	NA	ORR	79.1%	14	37	43/60%	22 (1–43)	16
Sougklakos I [16]^/^2011	Greece/2007–2010	-	Pros-, case series	30	16/14	64	NA	30/0^d^	NA	ORR	70%	11^e^	NR	16/57%	15.1 (NA)	14
ERIC ASSENAT [17]/2011	France/2006–2008	ERBIRINOX	Pros-, case series	42	22/20	60 (32–76)	30/12^a^	24/16/2^d^	NA	CRR	80.90%	9.5	24.7	15/NA	18.1 (0.4–39.6)	16
Z Saridaki [18]/2012	Greece/2007–2010	-	Pros-, case series	30	14/16	64 (36–70)	22/8^a^	NA	NA	ORR	70%	10.2^e^	30.3	16/62%	31 (13–37)	16
Folprecht, G [19]/2013	Germany/2014–2018	CELIM2	Pros-, cohort	28	NA	NA	NA	28/0/0^d^	28/0/0	ORR	86%	15	55	NA	NA	22
Fornaro, L [20]/2013	Italy/2010–2011	TRIP	Pros-, case series	37	21/16	63 (33–72)	26/11^a^	37/0/0^d^	37/0/0	ORR	89%	11.3	NR	12/75%	17.7 (NA)	16
Bendell, J. C [21]^*^/2016	USA/NA	-	Pros-, case series	15	13/2	55 (39–70)	NA	8/0/7^d^	NA	ORR	60%	13.3	NR	15/67%	15 (NA)	16
PietrantonioF [22]/2017	Italy/NA	-	Pros-, case series	31	NA	NA	NA	NA	NA	ORR	87%	17.8	62.5	31/84%	48 (NA)	14
Cremolini, C [23]/2018	Italy/2011–2015	MACBETH	Pros-, multi-center, cohort	116	82/34	59.5 (53–67)	95/21^b^	116/0/0	116/0/0	10m PFR	71.60%	10.1	33.2	52/51.9%	44 (30.5–52.1)	24
D Modest [24]/2019	Germany/NA	VOLFI	RCT, multi-center	63	41/22	58 (31–76)	53/10^b^	63/0/0	43/7/13	ORR	87.3%	9.7	35.7	NA	44.2 (NA)	14
Ogata, T [25]./2019	Japan/2014–2017	-	Retro-, cohort	17	NA	60	14/3^b^	17/0/0	NA	ORR	100%	13.1	NR	NA	18.4 (NA)	21
E Samalin [26]^*^/2019	NA	ESTER	Retro-, case series, multi-center	70	43/27	58.7 (40.4–74)	57/13^b^	70/0/0	NA	PFS	85.70%	13.3	48.5	68/79.5%	49.2 (1.2–135)	13
Deng, Y [27].^*^/2020	China/2014–2019	FOCULM	Pros-, case series, multi-center	67	58/9	52 (28–70)	66/1^b^	67/0/0	67/0/0	Rate of NED	95.50%	15.5	NR	67/46.3%	22.6 (NA)	16
Akihito Tsuji [28]/2021	Japan/2015–2019	DEEPER	RCT	173	NA	65	NA	173/0/0	NA	mDpR	69.10%	12.7	37.6	NA	NA	22

^*^All included patients were diagnosed as liver-limited colorectal cancer; ^a^colon/rectum, ^b^left/right, ^c^14 patients with cetuximab, 3 patients with panitumumab; ^d^only KRAS; ^e^time to progression, TTP; *wt*, wild-type, *mt*, mutant-type, *ORR* objective response rate, *mPFS* median progression-free survival, *mOS* median overall survival, *CLM* colorectal liver-limited metastases, *R0RR* R0 resection rate, *mfollow-up* median follow-up, *MINORS* methodological index for non-randomized studies, *pros-* prospective study, *retro-* retrospective study, *NA* not available, *NR* not reached, *CRR* complete response rate, *mDpR* median depth of response

The primary outcomes were ORR and R0RR in liver-limited mCRC patients. The secondary outcomes were mPFS, mOS, and grade 3/4 toxicity rate. ORR was defined as the ratio between the number of patients achieving an objective response (complete or partial response) and the total number of patients.

### Quality assessment

Because both case series and cohort studies were included in this meta-analysis, the Methodological Index for Non-Randomized Studies (MINORS) was used by two independent authors (H Wang and SQ Zhang) to assess the quality of the studies. The MINORS comprises 12 methodological items with a maximum score of 24. The first eight items (i.e., a clearly stated aim, inclusion of consecutive patients, prospective collection of data, endpoints appropriate to the aim of study, unbiased assessment of the study endpoint, follow-up period appropriate to the aim of the study, loss to follow-up less than 5%, prospective calculation of the study size) apply to both comparative and noncomparative studies, whereas the remaining four items (i.e., an adequate control group, contemporary groups, baseline equivalence of groups, adequate statistical analyses) apply only to comparative studies. A score lower than 10 for a case-series study or lower than 16 for a cohort study indicates low quality, and studies with such scores were excluded. The MINORS scores of the included studies are shown in Table 1. None of the studies was excluded on the basis of their score. Details on the MINORS score of each study are presented in Table 2.

**Table 2:** MINORS checklist for included studies

Study	Garufi	Sougklakos	ASSENAT	Saridaki	Folprecht	Fornaro	Bendell	Pietrantonio	Cremolini	Modest	Ogata	Samalin	Deng	Tsuji
*Methodological items for non-randomized studies*														
A clearly stated aim	2	2	2	2	2	2	2	2	2	2	2	2	2	2
Inclusion of consecutive patients	2	2	2	2	2	2	2	2	2	2	2	2	2	2
Prospective collection of data	2	2	2	2	2	2	2	2	2	2	1	1	2	2
Endpoints appropriate to the aim of study	2	2	2	2	2	2	2	2	2	2	2	2	2	2
Unbiased assessment of the study endpoint	2	2	2	2	2	2	2	2	2	2	2	2	2	2
Follow-up period appropriate to the aim of the study	2	2	2	2	2	2	2	2	2	2	2	2	2	2
Loss to follow up less than 5%	2	2	2	2	2	2	2	2	2	2	2	2	2	2
Prospective calculation of the study size	2	0	2	2	0	2	2	0	2	0	0	0	2	0
*Additional criteria for comparative studies*														
An adequate control group	-	-	-	-	2	-	-	-	2	-	2	-	-	2
Contemporary groups	-	-	-	-	2	-	-	-	2	-	2	-	-	2
Baseline equivalence of groups	-	-	-	-	2	-	-	-	2	-	2	-	-	2
Adequate statistical analyses	-	-	-	-	2	-	-	-	2	-	2	-	-	2
Total	16	14	16	16	22	16	16	14	24	14	21	13	16	22

The items are scored 0 (not reported), 1 (reported but inadequate), or 2 (reported and adequate). The global ideal score being 16 for non-comparative studies and 24 for comparative studies

### Statistical analysis

Statistical analyses were performed using the meta package of R 4.1.2 software. After data evaluation, we chose Freeman-Tukey double arcsine transformation to process the data presented as proportions with ORR, R0RR, and any grade 3/4 toxicity rate. We then aggregated using the inverse variance random-effect method (DerSimonian–Laird estimate). CIs for the individual studies were calculated based on the Clopper-Pearson interval method. Cochrane’s *Q* test and Higgins *I*-squared statistic were used to assess the heterogeneity of the included trials. *P*<0.1 in the *Q* test or *I*^2^>50% suggested significant heterogeneity. When significant heterogeneity was observed, the random effects model was used. Otherwise, the fixed effects model was adopted to evaluate the 95% CI. The sensitivity analysis was applied to explore the origin of heterogeneity. Publication bias was assessed by a visual inspection of the funnel plot, and the possibility of publication bias was assessed by Egger’s test.

## Results

### Search results and study characteristics

**Fig. 1:**
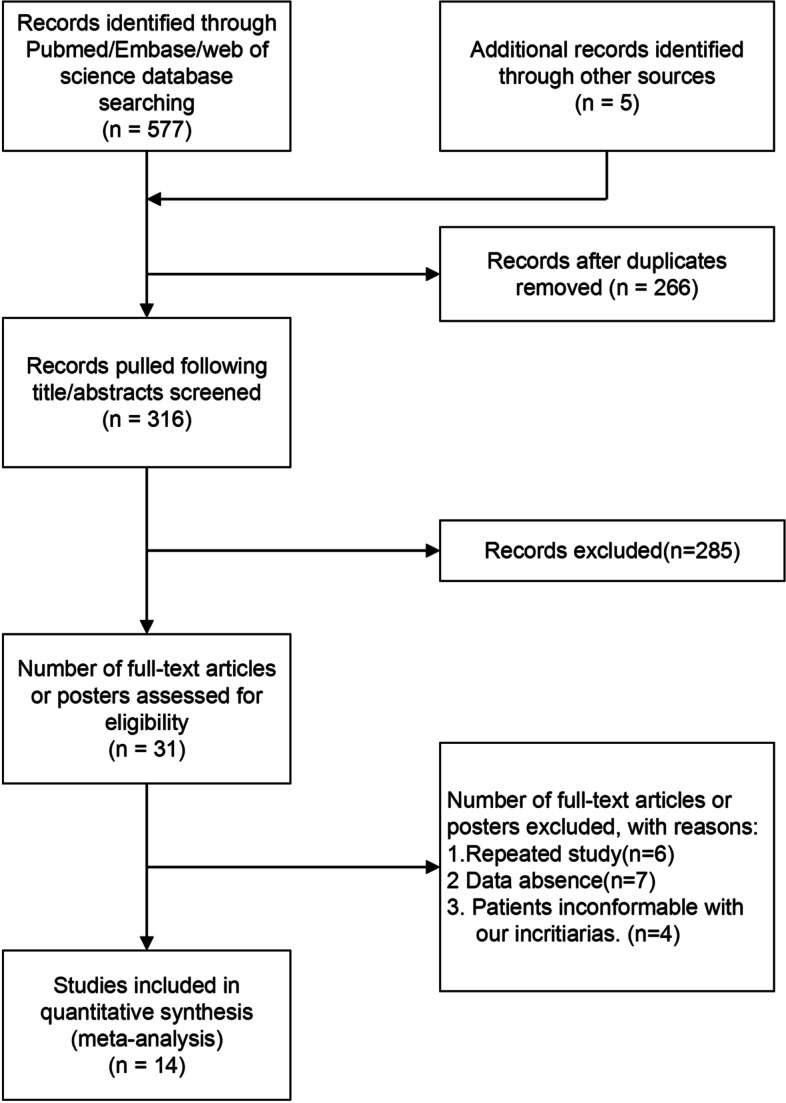
Flow diagram of literature search and study selection

A flow chart of the search strategies and reasons for exclusion is shown in Fig. 1.

In total, 577 potential articles were initially identified through the database, 266 articles were eliminated because of duplicate data, and 285 articles were removed after screening the title and abstract. The remaining 31 articles were retrieved for further assessment. Finally, 14 studies comprising 762 patients with mCRC were included in this meta-analysis. Among them, five studies were only described in conference abstracts.

The characteristics and study quality of all the included studies were evaluated by the MINORS and are presented in Table 2. Twelve studies were prospective, four were two-arm comparative cohort studies, ten were prospective/retrospective case series, four were multi-center investigations, and five were available only as conference abstracts or posters. A total of 762 patients were included across 14 studies. The median age range was 52–64 years (available in 11 studies), and the overall sex distribution was 338/175 (10 studies). All patients had an Eastern Cooperative Oncology Group (ECOG) performance status score of 0–1. RAS and BRAF mutations were detected in 701 and 311 patients, respectively. Among the 701 patients who accepted the RAS mutation test, 157 were KRAS wild-type, 390 were RAS wild-type (all tests were amended to the RAS test after the FDA changed its instructions for cetuximab), and 23 were KRAS mutants (all participated before the discovery of the negative role played in anti-EGFR agent treatment). Tumor primary locations were presented in 316 patients from 5 studies: 48 patients with tumors in the colon (side unspecified), 35 patients with tumors in the right colon, and 233 patients with tumors in the left colon or rectum. All patients were treated by a triplet chemotherapy (combination of 5-fluorouracil, irinotecan, and oxaliplatin/capecitabine) plus anti-EGFR agents (cetuximab or panitumumab). Details of the regimen in each study are shown in Table 3. Median cycles of chemotherapy were reported in eight studies with a range of 1–12. The metastasis location of 321 patients from 10 studies was limited to the liver; secondary R0RRs of these patients were reported in nine studies. In the four two-arm comparative studies, the treated arms included mFOLFOXIRI alone, mFOLFOXIRI plus anti-VEGF, and doublet chemotherapy (FOLFOX or FOLFIRI) plus anti-EGFR (one study).

**Table 3:** Summary of dose used in studies

Study	Treatment/median cycles (range)	Dose				Dose reduction or dose intensity
		Anti-EGFR	L-OHP (mg/m^2^)	CPT-11 (mg/m^2^)	5-FU (mg/m^2^)	
C Garufi/2010 [14]	Cet+Chrono-IFLO/6 (3–15)	Cet (400mg/m^2^ initial, 250 weekly mg/m^2^)	80	130	2400	L-OHP:60mg/m^2^ CPT-11:110mg/m^2^ 5-FU:2200mg/m^2^
Sougklakos, I./2011 [16]	Cet+FOLFOXIRI/NR	Cet 500mg/m^2^	65	150	600+400 (bolus)	NA
ERIC ASSENAT./2011 [17]	Cet+FOLFIRINOX/9 (1–12)	Cet (400 initial/250 weekly)	85	180	2400+400 (bolus)	76%required dose reduction, overall dose intensity was >90%
Z Saridaki/2012 [18]	Cet+FOLFOXIRI/12 (1–16)	Cet 500mg/m^2^	65	150	1200+400 (bolus)	NA
Folprecht, G./2013	Cet+FOLFOXIRI/NR	Cet 500mg/m^2^	85	125	3200	NA
Fornaro, L./2013 [20]	Pan+FOLFOXIRI/11 (3–16)	Pan 6mg/kg	85	150	3000	Relative dose intensity: L-OHP 75%, CPT-11 74%, 5-FU 76%
Bendell, J. C./2016 [21]	Pan+FOLFOXIRI/NA	Pan 6mg/kg	85	125	3200	NA
Pietrantonio, F/2017 [2017]	Cet+COI-E/NA	500mg/m^2^	85	180	1000 twice d2-5^b^	NA
Cremolini, C./2018 [23]	Cet+FOLFOXIRI/8 (6–8)	Cet 500mg/m^2^	85	130	2400	NA
D Modest/2019 [24]	Pan+FOLFOXIRI/11 (2–12)	Pan 6mg/kg	85	165	3200	NA
Ogata, T./2019 [25]	Cet+FOLFOXIRI/7 (1–14) Pan+FOLFOXIRI/12 (9–12)	Cet (400mg/m^2^ initial, 250 weekly mg/m^2^)	85	125/150/165^a^	3200	NA
E. Samalin/2019 [26]	Cet+FOLFIRINOX/10 (2–12)	NA	NA	NA	NA	NA
Deng, Y./2020	Cet+mFOLFOXIRI/7 (4–12)	Cet 500mg/m^2^	85	165	2800	Relative dose intensity: L-OHP 96%, CPT-11 96%, 5-FU 96%
Akihito Tsuji/2021 [28]	Cet+mFOLFOXIRI/10 (1–12)	Cet 500mg/m^2^	85	150	2400	NA

^a^Modified on UGT1A1 status; ^b^capecitabine; *Cet*, cetuximab; *Pan*, panitumumab; *CPT-11*, irinotecan; *L-OHP*, oxaliplatin; *5-FU*, 5-fluorouracil; *mFOLFOXIRI*, modified FOLFOXIRI; *COI-E*, irinotecan+oxaliplatin+capecitabine; *NA*, not available

### Primary outcomes

#### Objective response rate and R0 resection rate

**Fig. 2:**
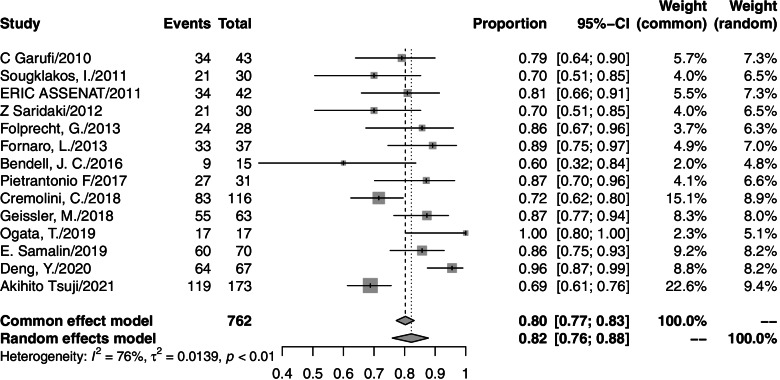
Forest plot of ORR in mCRC patients treated with triplet chemotherapy plus an anti-EGFR. ORR, objective response rate; mCRC, metastasis colorectal cancer

**Fig. 3:**
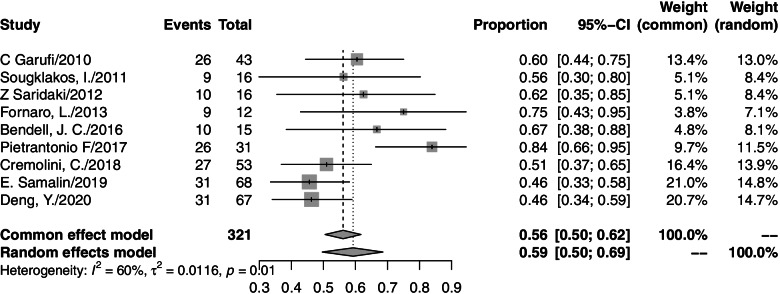
Forest plot of R0RR in CLM patients treated with triplet chemotherapy plus an anti-EGFR. R0RR, R0 resection rate; CLM, colorectal liver metastasis

A total of 763 patients from 14 studies were analyzed. The ORR from individual studies ranged from 60 to 100%. The pooled ORR was 82% (95% CI=76–88%, *I*^2^=76%; Fig. 2), and a random effects model was used. The responses to all included studies were evaluated by Response Evaluation Criteria in Solid Tumors (RECIST) criteria. Among the 14 studies, 9 studies presented the secondary R0RR in patients diagnosed with unresectable colorectal liver metastases (CLM). After accepting triplet chemotherapy plus an anti-EGFR agent therapy, 179 out of 321 CLM patients achieved R0RR. The range of R0RR was 46.3 to 84%, and the pooled R0RR was 59% with a random effects model (95% CI=50–69%, *I*^2^=60%). The forest plots of ORR and R0RR are shown in Figs. 2 and 3, respectively.

### Secondary outcomes

#### Median progression-free survival and median overall survival

The mPFS and mOS were obtained in 14 and 9 studies, respectively. The median PFS ranged from 9.5 to 17.8 months, and the mOS ranged from 24.7 to 62.5 months (Table 1).

### Grade 3/4 adverse events

**Fig. 4:**
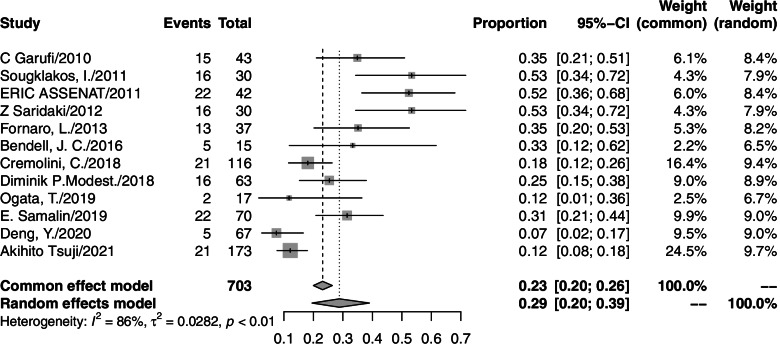
Forest plot of grade3/4 diarrhea in mCRC patients treated with triplet chemotherapy plus anti-EGFR. ORR, objective response rate; mCRC, metastatic colorectal cancer

**Fig. 5:**
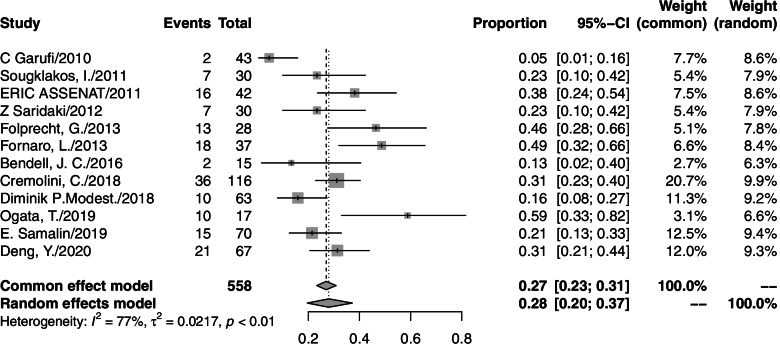
Forest plot of grade3/4 neutropenia in mCRC patients treated with triplet chemotherapy plus anti-EGFR. ORR, objective response rate; mCRC, metastatic colorectal cancer

**Fig. 6:**
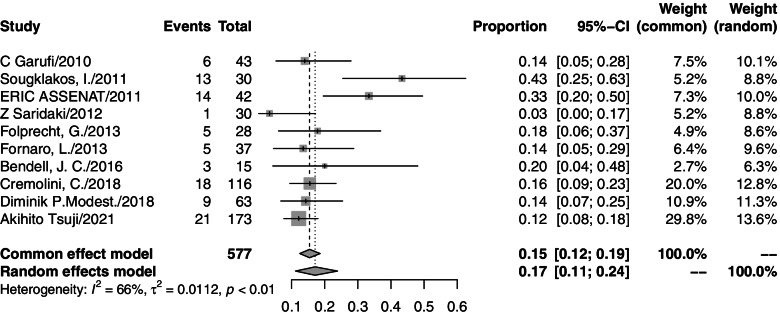
Forest plot of grade3/4 skin toxicity in mCRC patients treated with triplet chemotherapy plus anti-EGFR. ORR, objective response rate; mCRC, metastatic colorectal cancer

Thirteen studies reported grade 3/4 adverse events. In these studies, 648 grade 3/4 adverse events were observed among 731 patients who accepted triplet chemotherapy plus anti-EGFR agent therapy. One study reported a treatment-related toxic death related to neutropenic septicemia. The grade 3/4 toxicities described the most were diarrhea (174/648), neutropenia (157/648), and skin toxicity (95/648). The pooled rates were 29% (95% CI=20–39%, *I*^2^=86%), 28% (95% CI=20–37%, *I*^2^=77%), and 17% (95% CI=11–24%, *I*^2^=66%), respectively. A forest plot of each is shown in Figs. 4, 5, and 6. Toxicity profiles were not completed in five studies that were only available as a conference abstract. Most of the side effects were treatable. There were three studies that reduced the dose for a high incidence of adverse events (two because of diarrhea; one because of febrile neutropenia). All of them observed a significant decrease in toxicity after reduction. The toxicity profile is presented in Table 4.

**Table 4:** Summary of grade 3/4 toxicity in patients treated by triplet chemotherapy plus anti-EGFR agents

Author	Total	Neutropenia	FN	Thrombopnia	Diarrhea	Nausea/vomiting	Anorexia	Stomatitis	Asthenia	Skin toxicity	Neurotoxicity	HFS	Hypomagnesemia	Others
C Garufi	43	2	-	-	15	4	-	-	5	6	-	-	-	3
Sougklakos, I^a^	30	7	NA	NA	16	NA	NA	NA	NA	13	5	NA	NA	NA
ERIC ASSENAT	42	16	2	5	22	4	10	-	13	14	8	4	-	4
Z Saridaki	30	7	1	2	16	5	-	3	-	1	-	1	-	1
Folprecht, G^a^	28	13	NA	NA	NA	NA	NA	NA	NA	5	NA	NA	NA	NA
Fornaro, L	37	18	2	-	13	5	-	5	10	5	3	1	5	-
Bendell, J. C	15	2	-	1	5	1	2	-	3	3	-	1	1	2
Cremolini, C	116	36	4	-	21	3	4	7	11	18	-	-	-	-
D Modest	63	10	-	-	16	6	-	6	5	9	2	4	3	17
Ogata, T	17	10	-	-	2	1	-	-	-	-	-	-	2	-
E. Samalin^a^	70	15	2	NA	22	NA	NA	NA	NA	NA	NA	NA	NA	NA
Deng, Y	67	21	2	1	5	1	-	1	1	-	3	-	-	6
Akihito Tsuji^a^	173	NA	NA	NA	21	NA	NA	NA	NA	21	NA	NA	7	NA
Total	731	157	13	9	174	30	16	22	48	95	21	11	18	33

^a^Toxicity profile uncompleted; *FN* febrile neutropenia, *HFS* hand/foot syndrome, *NA* not available

### Publication bias

**Fig. 7:**
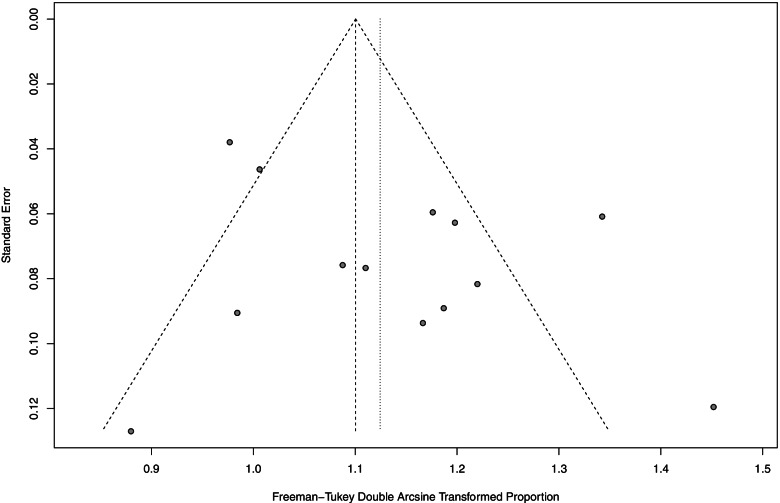
Funnel plot of ORR in mCRC patients treated with triplet chemotherapy plus anti-EGFR. ORR, objective response rate; mCRC, metastatic colorectal cancer

**Fig. 8:**
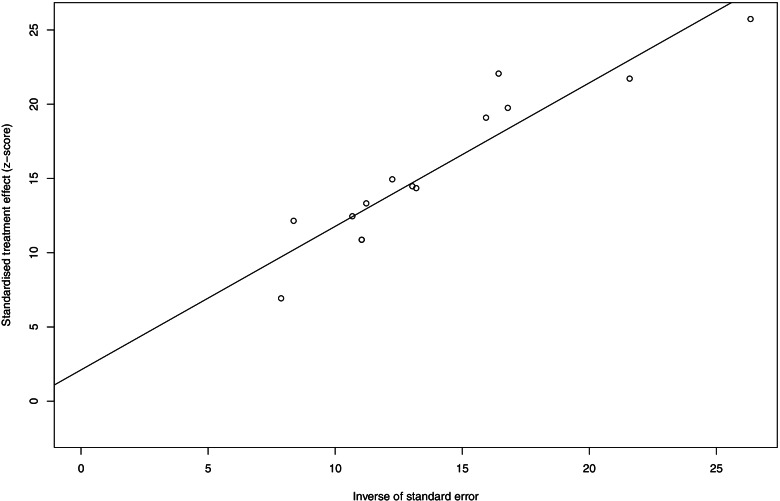
Egger’s test of ORR in mCRC patients treated with triplet chemotherapy plus anti-EGFR. ORR, objective response rate; mCRC, metastatic colorectal cancer

As shown in Figs. 7 and 8, the possible publication bias of the included studies was assessed by the funnel plot test and Egger’s linear regression test. No significant publication bias was detected from statistical tests based on ORR (*t*=1.33, *P*=0.2083).

## Discussion

To the best of our knowledge, our study is the first meta-analysis evaluating the efficacy of triplet chemotherapy plus anti-EGFR agent therapy. Nevertheless, there are multiple reviews and studies of this topic, which implies that researchers have devoted considerable attention to this important issue. Our meta-analysis combined the outcomes of 762 mCRC patients treated with triple chemotherapy plus anti-EGFR therapy from 14 studies, indicating that the treatment can improve the ORR to 82% and improve the secondary R0 rate without increasing the G3/4 adverse effect.

Despite the large number of studies comparing the efficacy of different regimens in mCRC first-line treatment, the optimal therapy is still controversial [29, 30], especially in CLM patients or potentially resectable mCRC patients who may need more intensive treatment to achieve distinct tumor shrinkage to purchase the chance of secondary surgery [31]. Since both FOLFOXIRI and anti-EGFR target therapy were considered to be related to more rapid tumor responses, we conducted this meta-analysis to assess the efficacy and safety of their combination.

In our meta-analysis, the pooled ORR of patients who accepted triplet chemotherapy plus anti-EGFR therapy was 82% (95% CI=76–88%, *I*^2^=76%), ranging from 60 to 100%. For comparison, we summarized several meta-analyses of important studies in mCRC treatment. Details are presented in Table 5. This result clearly dominates the efficacy of all the other treatments. This indicates that triplet chemotherapy plus anti-EGFR therapy conspicuously increases the chance of secondary resection in mCRC patients. The same advantages can be observed in other outcomes, such as the R0RR of CLM, mPFS, and mOS. A possible explanation of such a remarkable improvement is discussed below.

**Table 5:** Summary of related meta-analysis for a first-line treatment of mCRC

Meta-analysis	Included studies	Treatment	ORR	mPFS (month)	mOS (month)	SRR	Grade3/4 toxicity
Cremolini C [32]	CHARTA OLIVIA STEAM TRIBE TRIBE2	bev+3-CT vs bev+2CT^a^	64.5 vs 53.6% OR 1.57, *P*<0.001	12.2 vs 9,9 HR:0.74, *P*<0.001	28.9 vs 24.5 HR:0.81, *P*<0.001	16.4 vs 11.8% OR 1.48, *P*=0.007	Neutropenia 45.8 vs 21.5%; *P*<0.001 FN 6.3 vs 3.7%; *P*=0.019 Diarrhea 17.8 vs 8.4%; *P*<0.001
C Bokemeyer [33]	CRYSTAL OPUS	Cet+2CT vs 2CT	60.7 vs 40.9% OR 2.27, *P* <0.0001	10.9 vs 7.7 HR 0.64, *P*<0.0001	24.8 vs 21.1 HR 0.84, *P* =0.0048	NA	NA
F Pietrantonio [34]	Valentino TRIBE TRIBE2 STEAM CHARTA.	pan+3CT vs bev+3CT	73 vs 77% OR 0.79, *P* =0.4	11.4 vs 13.3 HR 0.83, *P* =0.11	30.3 vs 33.1 HR 0.8, *P* =0.14	22 vs 18% *P* =0.51	Neutropenia 26 vs 48%; *P* =0.001 Diarrhea 14 vs 6%; *P* =0.82 Febrile stomatitis 8 vs 6%; *P* =0.67
G Tomasello [35]	11 studies	Bev+3CT	69% (95%CI, 65–72%)	12.4 (95%CI,10-14.3)	30.2 (95%CI,26.5-33.7)	36.6% (95%CI,24.6%-50.5%)	NA

^a^All KRAS and BRAF wild type; *ORR* Objective response rate, *SRR* Secondary resection rate, *mPFS* Median progression-free survival, *mOS* Median overall survival, *2CT* Doublet chemotherapy, *3CT* Triplet chemotherapy, *FN* Febrile neutropenia

### RAS and BRAF mutation status

KRAS codons 12 and 13, 61, HRAS, NRAS, and BRAF V600E mutations are the most important and widely studied biomarkers for anti-EGFR agents. Both RAS and BRAF mutations have been proven to be prognostic factors associated with worse survival [36]. Because anti-EGFR target therapy is not recommended for patients with RAS or BRAF mutations, there were notable differences in patient baseline gene status selection in the included studies. In our meta-analysis, among the 701 patients who accepted the RAS mutation test, 157 were KRAS wild-type, 390 were RAS wild, and three were KRAS mutants. Among the 311 patients who accepted the BRAF mutation test, 291 were wild-type, and seven were mutant. It is assumed that the impressive efficacy was associated with the large number of RAS and BRAF wild-type patients.

### Primary tumor side

There is now a consensus that the primary tumor side of mCRC is biologically distinct [37]. The CALGB/SWOG 80405 study presented a significantly longer mPFS and mOS of the left side than the right side (mOS 33.3 months vs. 19.4 months, *P*<0,001) [38]. Subgroup analysis showed that in left-side-originated mCRC patients, compared with doublet chemotherapy plus bevacizumab, the combination of doublet chemotherapy and cetuximab was associated with a significantly longer mOS (36 vs. 31.4 months, *P*=0.018). This advantage was not reflected in the right-side originated mCRC patients; the mOS of cetuximab-arm and bevacizumab-arm were 16.7 vs. 24.2 months, *P*=0.065 [39]. The same tendency was found in the sub-analysis of trials FIRE-3 and PEAK [9, 40]. Based on this evidence, guidelines recommended treatment separately according to the primary tumor side. Anti-EGFR-targeted therapy is only recommended in left-side colon cancer combined with doublet chemotherapy, including FOLFOX, FOLFIRI, and XELOX [41]. In our meta-analysis, among the 762 patients included, tumor primary location was reported for 316 patients from five studies, including 48 patients with tumors in the colon, 35 patients with tumors in the right colon, and 233 patients with tumors in the left colon or rectum. Thus, a large proportion of left colon or rectal cancer patients may be one reason for our high ORR.

### R0 resection rate and conversion therapy

Conversion therapy is a standard therapy for mCRC patients, especially for patients with CLM. Recent studies verified that CLM is a particular type of mCRC that is heterogeneous to other mCRC patients [37]. Convincing evidence has proven that the R0RR of CLM can significantly increase the 5-year survival rate and mOS [42]. This inspiring discovery reflects the importance of seeking optimal treatment for the appropriate patients. The new treatment goal of CLM is currently to maximize the possibility of eradicating all LM lesions, which require rapid and distinct tumor shrinkage [43]. In our meta-analysis, the pooled R0RR in CLM patients was 60% with a random effects model (95% CI=49–70%, *I*^2^=69%). Among the 14 included studies, five studies were designed specifically to evaluate the R0RR of CLM after treatment with triplet chemotherapy plus anti-EGFRs agent, and the R0RR ranged from 60 to 84%. Despite these encouraging results, there were an additional 14 patients from two studies who accepted R1 resection (two patients) or R2 resection (12 patients). Other important indices for conversion therapy include early tumor shrinkage and depth of response (DpR), which were verified as capable of predicting treatment outcomes and cetuximab efficacy [44, 45]. Two studies used these indices as end points. The FOCULM study demonstrated that compared with mFOLFOXIRI alone, the addition of cetuximab improved the DpR from 44 to 56.1% (*P*=0.012), and the overall resection rates were 55.2% and 29.4%, respectively [27]. Consistent results of R0RR were presented in the POCHER trial (60%, 95% CI=45.8–75.1%), and another study conducted by E Samalin (57.4%) [14, 26]. All the results mentioned above demonstrated that the use of triplet chemotherapy plus the anti-EGFR agents significantly increased the possibility of the R0 resection of CLM patients, indicating that this treatment is preferable for CLM patients. It is important to identify the possible molecular and genetic markers of the patients who may benefit most from the treatment. As evidence has shown that anti-EGFR treatment is more effective in left-side colorectal cancer [46], it is safe to hypothesize that RAS and BRAF wild-type patients with liver metastatic left-sided cancer may possibly be the selected group to benefit most from triplet chemotherapy plus anti-EGFR agent therapy. We should acknowledge the small sample size and low quality of the included studies. This hypothesis needs to be tested in large-scale RCTs.

### Toxicity and dose adjustment

The most commonly observed adverse event in our study was diarrhea (174/648), followed by neutropenia (157/648) and skin toxicity (95/648). The toxicity profile was slightly different from that of triplet chemotherapy plus anti-VEGF therapy, in which neutropenia was the most frequently observed grade 3/4 adverse event, with an incidence of 45.8%, and the incidence of diarrhea was only 17.8% [30]. In our meta-analysis, the pooled rates were 29% (95% CI=20–39%, *I*^2^=86%), 28% (95% CI=20–37%, *I*^2^=77%), and 17% (95% CI=11–24%, *I*^2^=66%) for diarrhea, neutropenia, and skin toxicity, respectively. Three studies reported a dose reduction after a high incidence of toxicity, mainly because of diarrhea and neutropenia. After a dose adjustment, all studies considered the side effects manageable through symptomatic measures. The detailed dosage of each drug is presented in Table 2. Two studies pointed out that dose reduction did not influence efficacy. The results should be interpreted discreetly due to the retrospective design and small sample size. It should also be noted that nearly all included patients had an ECOG performance score of 0–1.

### Future prospects

With the development of surgery and toxicity management, the role of conversion therapy has become increasingly important in colorectal cancer treatment, especially in CLM patients. Thus, anti-EGFR plus triplet chemotherapy, which is considered to be able to increase the secondary surgery rate, has attracted researchers’ attention. However, despite the inspiring results mentioned above, several questions still should be answered. First, we discuss the dose and the treatment schedule of this therapy. It should be noted that the drug dose and treatment cycles in each clinical trial were not completely in accordance. Patients in several trials experienced dose reduction. In addition, there were also apprehensions about the adjuvant or second-line treatment selection. The second question relates to treatment endpoints. Since one of the important objectives of anti-EGFR plus triplet chemotherapy treatment was to enhance the R0RR, some clinical trials chose ETS and DpR as primary endpoints instead of the ORR, which is the most frequently used endpoint. These indices might be easier for readers to interpret. However, we should not neglect our ultimate purpose, which is to prolong overall survival. Thus, complete follow-up is necessary for the final evaluation of this treatment. Third, the optimal criteria for patient selection remain unclear. It remains unclear how we can identify suitable treatment for patients according to their gene status, original side, metastatic condition, and so on. Fortunately, there are several ongoing RCTs, including DEEPER (mFOLFOXIRI+cetuximab vs. mFOLFOXIRI+bevacizumab, NCT02515734), TRIPLETE (mFOLFOXIRI+panitumumab vs. mFOLFOX6+panitumumab, NCT03231722), and PANIRINOX (mFOLFIRINOX+panitumumab vs. mFOLFOX6+panitumumab, NCT02980510), which aim to compare the efficacy of different combinations of doublet or triplet chemotherapy plus different target agents, and their results may be helpful in this regard.

### Limitations

This study has several limitations that should be recognized. First, the eligible studies and the sample sizes of the included studies were relatively small. Heterogeneity between studies was relatively high, which may bias the results. Despite the utility of sensitivity analysis, the origin of heterogeneity could not be fully traced. Second, most of the studies were case series or retrospective studies, which may influence the accuracy of the results (especially in the assessment of adverse events). Third, a portion of the studies were only obtainable as conference abstracts. Despite efforts made to get in touch with authors for complete results, there remained crucial data uncollected. Fourth, the dosage of each study was not consistent; one of the included studies used capecitabine instead of 5-fluorouracil, affecting the outcome of the study and toxicity evaluation. Finally, this study was constrained to studies published in the English language only. Thus, the potential for publication bias cannot be ignored.

## Conclusion

Triplet chemotherapy plus anti-EGFR therapy seems to be capable of increasing the ORR of mCRC patients and R0RR of CLM patients. The toxicity of this treatment is manageable. High-quality RCT studies are required for further validation.

## Supplementary Information

**Additional file 1.**

## Data Availability

The datasets used and/or analyzed during the current study are available from the corresponding author on reasonable request.

## Abbreviations

mCRC: Metastatic colorectal cancer
anti-EGFR: Anti-epidermal growth factor receptor
ORR: Objective response rate
R0RR: R0 resection rate
mPFS: Median progression-free survival
mOS: Median overall survival
RCT: Randomized controlled trial
CLM: Colorectal liver metastasis
HR: Hazard ratio
CI: Confidence interval
RAS: Rat sarcoma
FDA: Food and Drug Administration
ASCO: American Society of Clinical Oncology
MINORS: Methodological Index for Non-Randomized Studies
ECOG: Eastern Cooperative Oncology Group
RECIST: Response Evaluation Criteria in Solid Tumors
DpR: Depth of response
TTP: Time to progression
wt: Wild-type
mt: Mutant-type
pros-: Prospective study
retro-: Retrospective study
NA: Not available
NR: Not reached
CRR: Complete response rate
Cet: Cetuximab
Pan: Panitumumab
CPT-11: Irinotecan
L-OHP: Oxaliplatin
5-FU: 5-Fluorouracil
*mFOLFOXIRI*: Modified FOLFOXIRI
*FN*: Febrile neutropenia
HFS: Hand/foot syndrome
*SRR*: Secondary resection rate
2CT: Doublet chemotherapy
3CT: Triplet chemotherapy
